# The involvement of NLRP3 inflammasome in CUMS-induced AD-like pathological changes and related cognitive decline in mice

**DOI:** 10.1186/s12974-023-02791-0

**Published:** 2023-05-10

**Authors:** Jia-Mei Li, Ting Hu, Xiao-Na Zhou, Ting Zhang, Jia-Hui Guo, Min-Yuan Wang, Yi-Lin Wu, Wen-Jun Su, Chun-Lei Jiang

**Affiliations:** 1 Department of Stress Medicine, Faculty of Psychology, Naval Medical University, Xiangyin Road 800, Shanghai, 200433 China; 2 Department of Neurology, Navy 971st Hospital of PLA, Minjiang Road 22, Qingdao, 266071 China

**Keywords:** NLRP3 inflammasome, Depression, Alzheimer’s disease, Cognitive decline, Neuroinflammation

## Abstract

**Background:**

Numerous studies have found that inhibiting the expression of NLRP3 inflammasome can significantly improve depressive-like behaviors in mice, but the research on its effect on cognitive decline in depression and its mechanism is still lacking. This study aimed to elucidate the role of NLRP3 inflammasome in cognitive decline in depression and explore the common neuro-immunological mechanisms of depression and Alzheimer’s disease (AD).

**Methods:**

Male C57BL/6 mice were subjected to chronic unpredictable mild stress (CUMS) for 5 weeks, treatment group was administered with the NLRP3 inhibitor MCC950 (10 mg/kg, i.p.), fluoxetine served as positive control. Then, the mice were assessed for cognitive behaviors and depression-like behaviors, and changes of microglia and neurons in hippocampus and levels of Aβ metabolic pathway and tau protein were measured. To explore the mechanism of NLRP3 activation on neurons, we performed in vitro studies using BV2 microglia and mouse primary neurons. Furthermore, we focused on the role of NLRP3 inflammasome in the function of neurons and the expression of AD pathological indicators.

**Results:**

CUMS induced depressive-like behaviors and cognitive decline in mice, which could be reversed by inhibiting NLRP3 inflammasome. MCC950, a specific NLRP3 inhibitor, alleviated CUMS-induced neuron injury and AD-like pathological changes, including the abnormal expression of Aβ metabolic pathway and the hyper-phosphorylation of tau protein. LPS (1 μg/mL) + ATP (1 mM) treatment activated the expression of NLRP3 inflammasome and IL-1β in vitro. In vitro experiment also proved that inhibiting the expression of NLRP3 inflammasome in microglia can restore the Aβ metabolic pathway to normal, decrease neuronal tau protein phosphorylation and protect neurons.

**Conclusions:**

Inhibition of NLRP3 inflammasome effectively alleviated CUMS-induced depressive-like behaviors and cognitive decline in mice, and inhibited the activation of AD physiological indicators.

**Supplementary Information:**

The online version contains supplementary material available at 10.1186/s12974-023-02791-0.

## Background

With the rapid development of economy and society, and changes in lifestyle, people are under increasing pressure from work and life. Long-term high levels of pressure and stress can increase the risk of developing anxiety, depression, and other mental disorders [1, 2]. Depression is one of the most common psychiatric diseases, characterized by low mood, thinking retardation, less motivation, and activity. Patients with major depression exhibit cognitive decline and a series of autonomic nervous dysfunction including insomnia, fatigue, loss of appetite, etc. [3]. Cognitive decline is one of the most common residual symptoms of depression, which is manifested as a decline in attention, executive function, working memory, and information processing speed [4, 5]. Most patients cannot obtain full recovery in cognitive function even in remission stage [6]. In addition, the cognitive decline of depression may also have an impact on the long-term neurological function of patients with depression. Depression can significantly increase the risk of AD late in life [7]. At present, the pathogenesis of cognitive decline in depression is still unclear. And there is currently no specific drug for the treatment of cognitive decline in depression.

A large number of clinical and basic studies have shown that depression and AD may share a common pathophysiological basis, and a variety of pathogenic factors are involved. Chronic inflammatory response plays a central role in the pathological mechanisms of both depression and AD, and may be the key pathological mechanism shared by depression and AD [8, 9]. The pro-inflammatory cytokine interleukin 1β (IL-1β) is one of the main mediators of neuro-inflammatory response. The level of IL-1β in peripheral blood of patients with depression is much higher than that of normal people. Recent findings have shown that the higher the level of IL-1β in peripheral blood, the worse the cognitive function of patients with depression [10]. The level of IL-1β in peripheral blood of AD patients is also significantly higher than that of normal people [11]. IL-1β can mediate inflammatory response-induced cognitive decline by inhibiting the long-term potentiation of the hippocampus and affecting hippocampal neurogenesis [12, 13].

The mature activation of IL-1β is dependent on its upstream molecule, the NLRP3 inflammasome. The inflammasome effector protein, Caspase-1, can cleave the IL-1β precursor Pro-IL-1β, making it into biologically active IL-1β which drives the pro-inflammatory response [14]. The NLRP3 inflammasome is an important component of the innate immune system. Cognitive impairment caused by various diseases is associated with the activation of NLRP3 inflammasome. The application of NLRP3 inflammasome inhibitors or RNA knockdown technology (siRNA-NLRP3) in cognitive impairment models can restore the expression of inflammasomes to normal and significantly improve cognitive function [15–17].

Numerous studies have found that inhibiting the expression of NLRP3 inflammasome can significantly improve depressive-like behavior in acute and chronic stress model mice [18, 19]. However, effect and mechanism of NLRP3 inflammasome on cognitive decline in depression remain to be elucidated. Besides, little is known about how depression increases the risk of Alzheimer's disease. There is also a lack of treatment drugs and objective diagnostic indicators for cognitive decline in depression in clinic practice. Thus, the study aimed to explore how NLRP3 inflammasome modulates cognitive decline in depression induced by chronic stress.

## Materials and methods

### Animals and treatments

A total of 36 adult male C57BL/6 mice (6 weeks old) with average weight of 22 g were obtained from Experimental Animal Center of Naval Medical University (Shanghai, China). All animals were habituated for 2-week acclimation with 1% sucrose solution (weight/volume) following previous study, and bred at consistent ambient temperature (22 ± 2 °C), on a 12-h light/dark cycle with free access to food and water. All mice were tagged with metal ear tags before formal experiments, and then randomly assigned to four groups on average, namely Control, CUMS, CUMS + MCC950, and CUMS + Flx group (n = 9 for each group). All procedures were conducted in accordance with the guidelines of Ethics Committee of the Naval Medical University.

MCC950 is a specific NLRP3 inflammasome inhibitor that crosses the blood–brain barrier and modulates the inflammatory response. In the experiment, NLRP3 inflammasome inhibitor MCC950 and selective 5-HT reuptake inhibitor Fluoxetine (Flx) were purchased from Selleck (#S7809, Houston, TX, USA) and MedChem Express (#LY110140, Monmouth Junction, NJ, USA), respectively. After dissolved in sterile 0.9% saline at 2 mg/mL concentration, MCC950 and fluoxetine were daily administrated at a dose of 10 mg/kg body weight (i.p.). Drug treatment or vehicle administrations began with the start of CUMS paradigm. For in vivo study, we prepared 0.1 μM, 1 μM, and 10 μM concentrations of MCC950.

### Chronic unpredictable mild stress (CUMS)

**Fig. 1:**
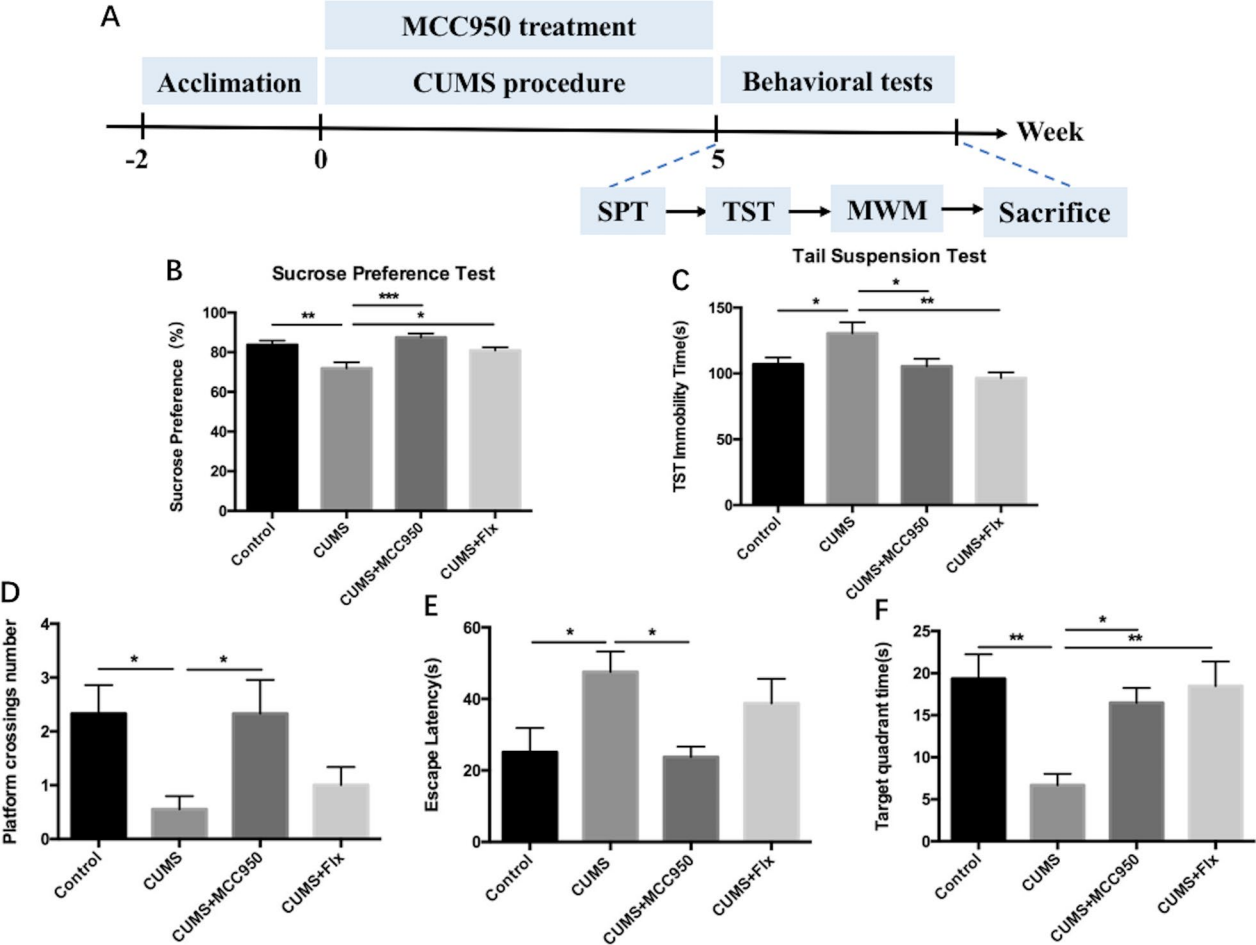
MCC950 improved CUMS-induced depressive-like behaviors and cognitive decline. **A** The sucrose preference in CUMS mice was lower than Control group, while MCC950 and fluoxetine could improve. **B** MCC950 and fluoxetine could reverse CUMS-induced prolonged tail suspension time. **C** MCC950 could reduce platform crossings numbers of CUMS mice, but fluoxetine could not. **D** MCC950, rather than fluoxetine, improved escape latency of CUMS mice. **E**,** F** The time of CUMS mice in the target quadrant was shortened, and both MCC950 and fluoxetine could relieve. The data were shown by mean and standard error (Mean ± SEM). (*n* = 9 for each group, *P < 0.05, **P < 0.01, ***P < 0.001)

Male mice were caged individually and subjected to the chronic unpredictable mild stress (CUMS) protocol. The CUMS paradigm was adapted from our past research, involving exposure to a series of mildly intense stressors in random order [20]. The stressors include 45° cage inclination for 18 h, reversal of the light–dark cycle for 24 h, restraint for 2–4 h, food or water deprivation for 20 h, 45 °C dry-heat stress for 10 min, bedding deprivations for 12 h, damp bedding for 16 h, cage vibration for 20 min, and swimming at 4 °C for 4 min. After 5-week CUMS, the paradigm was stopped. The timing of the processing schedules for CUMS and behavioral tests was shown in Fig. 1A. The behavioral tests were carried out after CUMS. And the mice were sacrificed immediately after tests.

### Sucrose preference test (SPT)

Sucrose preference test (SPT) was used to evaluate the depressive-like behaviors. Before measuring, mice were presented with two bottles of 1% (weight/volume) sucrose solution for the first week. The following week, mice were presented with two bottles choice: one bottle containing 1% sucrose solution, while the other containing tap water. Positions of two bottles were exchanged every day to eliminate the learning effect. According to previous literature [21], mice were individually housed, deprived of food and water for 18 h, and then provided with two bottles of the same size: one captaining 1% sucrose solution and another one containing clean tap water. The bottles were placed alternately to avoid a side bias. Fluid consumptions were evaluated after 1 h test. Sucrose preference was calculated as the percentage of sucrose solution consumption out of total liquid consumption.

### Tail suspension test (TST)

Tail suspension test is widely applied to evaluate the despair behavior in rodent model. Each mouse was suspended upside down by the end of its tail on the hook of a standard apparatus, PHM-300 tail suspension chamber (MED Associates Inc, St. Albans, VT). A 1.5 cm plastic tube was attached to the tail of the mouse to avoid mice climbing. After a 1-min period of adaption to the apparatus, the immobility time during the last 5-min suspension was recorded and analyzed using the software (Tail Suspension SOF-821, Med Associates Inc.) with a threshold of 0.5. To minimize the influence of smell, 75% ethanol was used to clean the inner walls of the device between each trial.

### Morris water maze (MWM)

Morris water maze was used to assess the cognitive functions, such as learning, memory, and spatial orientation of rodents. The Morris water maze consisted of a pool, 120-cm in diameter, filled with water and mixed whiter dye (temperature: 20 ± 2 °C) to 1 cm above the escape platform (6 × 6 cm). Plastic plates of different colors (yellow, green, blue, and red) and different geometries (square, triangle, circle, and pentagram) were suspended as visual references on four pillars next to the tank. The mice were trained for six consecutive days to find a platform hidden that submerged 1 cm below the water surface. In 5-day training period, the mice entered the pool in 4 directions (southwest, south, east, and northeast), looking for a platform located in the northwest position. During 60 s training, mice were considered to successfully find the platform if they stayed on it for 15 s. Otherwise, the mouse was manually placed on the platform for 15 s to remember the position. Each mouse was trained 1 time a day at each orientation in random order. On day 6, trajectory, fastest time to find the platform, number of times to cross the platform, and time spent in the original quadrant of the platform were recorded in the condition that mice entered the pool in southeast direction without platform hint.

### Sacrifice and sample collection

After the behavioral tests, all animals were anesthetized with isoflurane (RWD, Shenzhen, China) for tissue collection and brain perfusion. Three mice of each group were perfused for staining. The remaining mice were sacrificed under cervical dislocation. Hippocampus was collected or dissected immediately after mice sacrifice, and stored at − 80 °C freezer.

### Cell line culture and treatments

A murine microglia cell line BV2 was obtained from American Type Culture Collection (ATCC, Rockville, MD, USA). BV2 cells were divided into the following five groups: Control group, LPS + ATP group, LPS + ATP + 0.1 μM MCC950 group, LPS + ATP + 1 μM MCC950 group, and LPS + ATP + 10 μM MCC950 group (*n* = 4 for each group). Cells were maintained in Dulbecco’s modified Eagle’s medium (DMEM; #10569-010, Gibco, Grand Island, NY, USA) supplemented with 10% fetal bovine serum (FBS; #10099133C, Gibco, Grand Island, NY, USA) and 1% double antibody solution (#15240-096, Gibco, Grand Island, NY, USA) at 37 °C in an incubator with 5% CO_2_. When the cell density reached approximately 80%, DMEM was replaced by the serum-free DMEM high-glucose medium. After pre-treatment with MCC950 (0.1 μM, 1 μM, and 10 μM) for 2 h, cells were administrated with lipopolysaccharide (LPS, 1 μg/mL; #L2880, Sigma-Aldrich, Germany) for 6 h. Afterward, adenosine triphosphate (ATP, 1 mM; #10129ES03, Yesen, Shanghai, China) was added to continue culturing for 2 h.

### Primary neuron culture and co-culture

Primary neurons were isolated from the hippocampus of postnatal (~ 24 h old) C57/BL6 mice as previous research [22]. After meninges removed, trypsinization and mechanical disruption were utilized to dissociate the tissues into a single cell suspension. The cells were pelleted by centrifugation for 5 min at 1000 rpm. To prepare primary neurons, pelleted cells were re-suspended gently and plated in poly-lysine-coated (#A3890401, Gibco, Grand Island, NY, USA) 6-well plates. After 4 h, DMEM was replaced by Neurobasal medium (#21103-049, Gibco, Grand Island, NY, USA) containing B27 (#12587-010, Gibco, Grand Island, NY, USA) and 0.5% double antibody solution at 37 °C with 5% CO_2_. Then the resuscitated BV2 cells were plated on the upper chamber of transwell 6-well plate (Corning, USA), while primary neurons were plated on the bottom chamber. When the plated BV2 cells density reached approximately 80%, DMEM was replaced by the serum-free DMEM high-glucose medium. In this experiment, BV2 cells were divided into the following four groups: Control group, LPS + ATP group, MCC950 group, and LPS + ATP + MCC950 group. After pre-treatment with MCC950 (1 μM) for 2 h, cells were administrated with LPS and ATP, a procedure as same as the previous step. Upper chamber with treated BV2 cells was then transferred to transwell 6-well plate with primary neurons for co-cultures for 24 h. The grouping and name of primary neurons were the same name as BV2 group in the same plate (*n* = 3 for each group). Primary neurons were collected for Western blotting.

### Western blotting

Hippocampus samples and treated cells were homogenized with RIPA buffer (#P0013B, Beyotime, Shanghai, China), PMSF protease inhibitor (#ST506-2, Beyotime, Jiangsu, China), and PhosSTOP (#4906845001, Roche, Germany) as previously described. The homogenized lysates were added with 5 × loading buffer (#P0015, Beyotime, Shanghai, China), heated at 100 °C for 10 min and stored at − 20 °C. The western blot procedure was described as our previous research. In brief, the denatured protein samples were separated by 10% sodium dodecyl sulfate–polyacrylamide gel electrophoresis gels (Epizyme Biomedical Technology, Shanghai, China) and transferred to polyvinylidene fluoride membranes (Millipore, Bedford, MA, USA). After blocked with NCM Biotech blocking buffer (NCM Biotech, Suzhou, Jiangsu, China) for 10 min, the membranes were incubated with specific primary antibodies (Additional file 1: Table S1) at 4 °C overnight. Washed three times, the membranes were incubated with IRDye conjugated secondary antibodies (1:5000) (LI-COR, Inc., USA) at room temperature for 1 h. For visualizing and quantifying blotting bands, Odyssey Infrared Imaging System (LI-COR, Inc., USA) and ImageJ Software (NIH, Bethesda, MD, USA) were utilized.

### Immunofluorescence

Mice were perfused transcardially with ice-cold 0.9% saline followed by ice-cold 4% paraformaldehyde solution (#G1101, Servicebio, Wuhan, Hubei, China). The whole brains were then removed, post-fixed in 4% paraformaldehyde overnight. Then the brains were washed in fluid water, dehydrated in ethanol and xylene, and embedded in melted paraffin for 2 h. After coagulation in room temperature, the brains were cut at 5 μm. Prior to immunofluorescence, the slices were deparaffined in xylene and dehydrated in ethanol. Approximately 10 sections, including the hippocampus region, were collected from each brain. After blocked with 3% BSA buffer, slices were incubated with antibody against DCX (#sc-271390, Santa Cruz, Dallas, TX, USA) at 4 °C overnight, and then in the second antibody for 1 h at room temperature. Nuclei were stained by DAPI (Ruchuang, Shanghai, China) for 15 min at room temperature. Fluorescent image acquisition was conducted using an inverted fluorescence microscope (Carl Zeiss, Germany). The data were analyzed by ImageJ Software.

### Nissl stain

The brain slices were stained in toluidine blue (#G3668, Solarbio life science, Beijing, China) at 30 °C for 10 min. The following procedures were conducted as previous for dehydration and deparaffinization. After blocked with resinous mounting medium, image acquisition was conducted using an inverted fluorescence microscope and analyzed by ImageJ Software.

### Immunohistochemistry

Before immunohistochemistry, brain slices were blocked with hydrogen peroxide solution at room temperature for 20 min. Afterward, slices were incubated in in antibody against Aβ (#NBP2-13075, Novus, Littleton, CO, USA) at 4 °C overnight and horseradish peroxidase labeled second antibody at room temperature for 1 h. The chromogenic reaction was terminated by washing sections in PBS until slices’ fully stained. Stained tissue sections were then dehydrated in alcohol and counter-stained with hematoxylin. After differentiation, slices were promptly washed in PBS for 3 times and analyzed by ImageJ Software.

### Immunocytochemistry

After 72-h culture, primary neurons were fixed by ice-cold 4% paraformaldehyde solution for 15 min and then washed with ice-cold 0.01 M PBS. After permeabilization with 0.2% TritonX-100 (#P0096, Beyotime, Shanghai, China), fixed primary neurons were blocked by 10% fetal bovine serum at room temperature for 1 h. Blocked cells were incubated in primary antibody (anti-NeuN: 1:100, #ab177487, Abcam, Cambridge, UK) at 4 °C overnight, and then in the second antibody for 1 h at room temperature. Nuclei were stained by Hoechst33258 (#C1018, Beyotime, Shanghai, China) for 5 min at room temperature. Fluorescent image acquisition was conducted using an inverted fluorescence microscope. The data were analyzed by ImageJ Software.

### Statistical analysis

All data were presented as mean ± SEM and analyzed by GraphPad Prism 6.0 (GraphPad Software, Inc., La Jolla, CA, USA). One-way ANOVA was used for comparisons between groups, and Tukey multiple comparisons were used for post hoc tests. Differences considered statistically significant at *P* < 0.05.

## Results

### MCC950 improved depressive-like behaviors and cognitive decline induced by CUMS in mice

MCC950 is one of the most effective inhibitors of NLRP3 inflammasome so far. It inhibits the activation of inflammasome and the secretion of inflammatory cytokines by specifically inhibiting the second signal pathway of NLRP3 inflammasome [23]. To explore the role of NLRP3 inflammasome in cognitive decline in depression, mice were intraperitoneally injected with MCC950 at a dose of 10 mg/kg body weight.

As shown in Fig. 1, after 5 weeks of chronic stress, the sucrose preference scores of CUMS mice were lower than those of Control mice. MCC950 effectively increased sucrose preference scores of CUMS mice to normal, and fluoxetine, an antidepressant, had similar protective effect on mice. At the same time, the tail suspension time of mice in CUMS group was prolonged, while this could be improved by daily injection of MCC950 or fluoxetine. In Morris water maze experiment, mice in CUMS group had fewer times of crossing the platform than those in Control group. MCC950 could effectively increase the times of crossing the platform of CUMS mice, but fluoxetine could not improve. The escape latency of mice in CUMS group was longer than that in Control group, but MCC950 could shorten the time of searching platform for CUMS mice, while fluoxetine could not. Meanwhile, the time of CUMS mice in the target quadrant decreased, while both MCC950 and fluoxetine improved this index.

### MCC950 alleviated CUMS-induced neuron injury and microglia activation

**Fig. 2:**
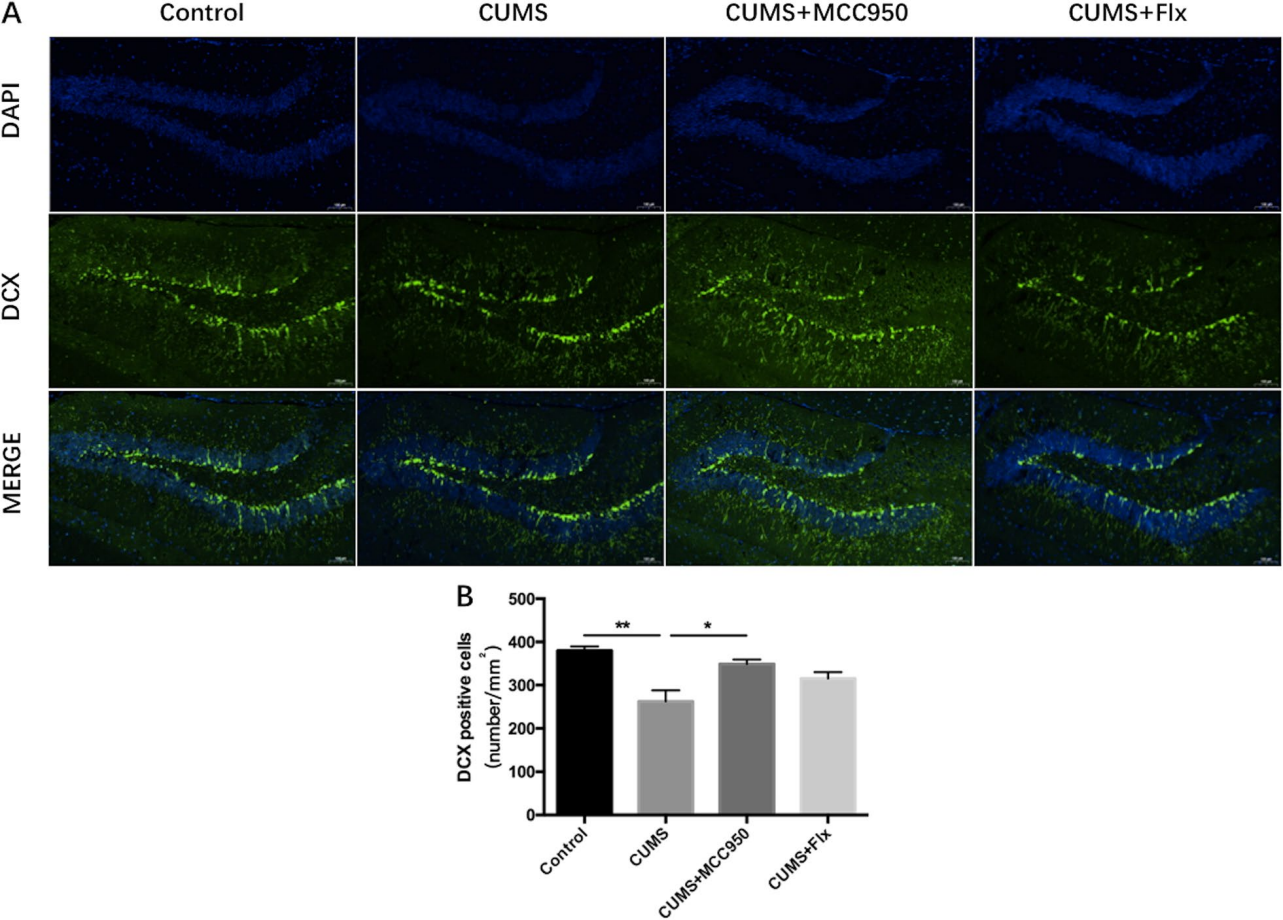
MCC950 alleviated the decrease of immature neurons induced by CUMS. **A** Immunofluorescence was used to detect the expression and distribution of the target protein. **B** CUMS induced the decrease of DCX positive cells in hippocampus, and MCC950 could significantly improve. The data were shown by mean and standard error (Mean ± SEM). (*n* = 3 for each group, *P < 0.05, **P < 0.01. Scale bar 100 μm)

Microtubules and related proteins, which are the main components of cytoskeleton, play an important role in the migration and differentiation of neurons [24]. Doublecortin (DCX) is a gene encoding microtubule binding protein, which is a marker of immature neurons [25]. We performed DCX immunofluorescence staining on hippocampus. As shown in Fig. 2, compared with the control group, the number of DCX positive cells in CUMS group decreased significantly (Fig. 2B; F_(3, 8)_ = 9.414, *P* = 0.0053; Tukey’s test: Control vs. CUMS, *P* = 0.0043). MCC950 could alleviate the decrease of DCX positive cells induced by CUMS (Tukey’s test: CUMS vs. CUMS + MCC950, *P* = 0.0238), but fluoxetine could not.

**Fig. 3:**
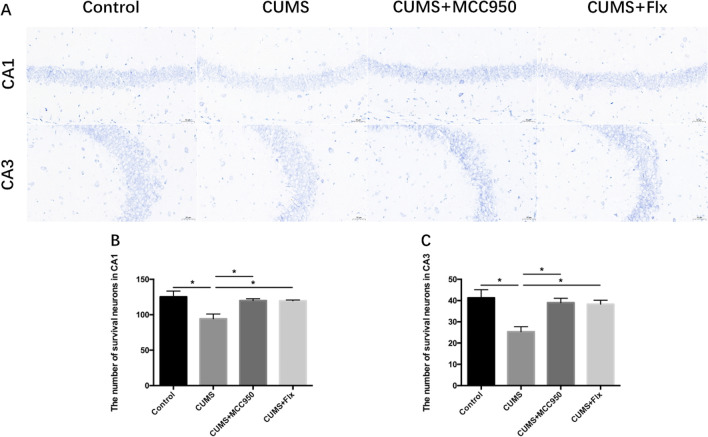
MCC950 reversed CUMS-induced decrease of Nissl staining positive cells. **A** Nissl staining in hippocampal CA1 and CA3 regions. **B** MCC950 and fluoxetine reversed CUMS induced decrease in the number of Nissl positive cells in CA1. **C** MCC950 and fluoxetine reversed CUMS induced decrease in the number of Nissl positive cells in CA3. The data were shown by mean and standard error (Mean ± SEM). (*n* = 3 for each group, *P < 0.05. Scale bar 50 μm)

Nissl staining is a common staining method to explore the morphology and pathology of nerve [26]. Nissl bodies, also known as tiger spots, are one of the characteristic structures of neurons. Nissl staining marks Nissl bodies located in neurons using the basophilic characteristics of Nissl bodies. The number and the integrity of morphological structure of Nissl bodies can reflect the functional activity of neurons [27]. The experimental results showed that the number of Nissl staining positive cells in CA1 area of hippocampus in CUMS group decreased significantly, while MCC950 and fluoxetine could improve (Fig. 3B; F_(3, 8)_ = 6.541, *P* = 0.0152; Tukey’s test: Control vs. CUMS, *P* = 0.0160; CUMS vs. CUMS + MCC950, *P* = 0.0414; CUMS vs. CUMS + Flx, *P* = 0.0440). Besides, chronic stress significantly reduced the number of Nissl staining positive cells in CA3 region, while MCC950 and fluoxetine could also alleviate, as shown in Fig. 3 (Fig. 3C; F_(3, 8)_ = 7.642, *P* = 0.0098; Tukey’s test: Control vs. CUMS, *P* = 0.0108; CUMS vs. CUMS + MCC950, *P* = 0.0251; CUMS vs. CUMS + Flx, *P* = 0.0322).

**Fig. 4:**
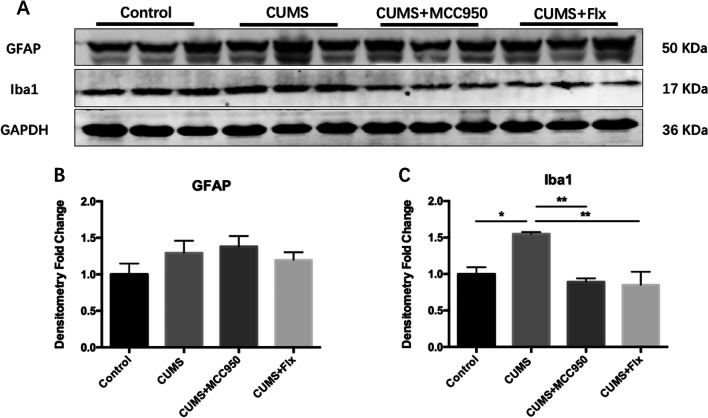
MCC950 alleviated the activation of microglia induced by CUMS. **A** The expression of target protein was detected by Western Blotting. **B** There was no significant difference in GFAP expression among groups. **C** CUMS induced the up-regulation of Iba1 expression in hippocampus, while MCC950 and fluoxetine intervention could significantly improve. The data were shown by mean and standard error (Mean ± SEM). (*n* = 3 for each group, *P < 0.05, **P < 0.01)

GFAP and Iba1 are the markers of astrocytes and microglia, respectively. GFAP is an important fibrin in mature astrocytes, which is involved in regulating the stability of cell structure [28]. Iba1 is expressed in numerous kinds of cells, but in the central nervous system, it is only expressed by microglia. When microglia are activated, the activity and expression of Iba1 increase, which leads to actin activity and takes part in membrane phagocytosis [28]. As shown in Fig. 4, there was no significant difference in GFAP expression in each group. However, CUMS significantly up-regulated the expression of Iba1 (Fig. 4C; F_(3, 8)_ = 9.513, *P* = 0.0051; Tukey’s test: Control vs. CUMS, *P* = 0.0248), while both MCC950 and fluoxetine could effectively inhibit the up-regulation of Iba1 induced by CUMS (Tukey’s test: CUMS vs. CUMS + MCC950, *P* = 0.095; CUMS vs. CUMS + Flx, *P* = 0.0067).

### MCC950 effectively inhibited CUMS-induced activation of NLRP3 inflammasome

**Fig. 5:**
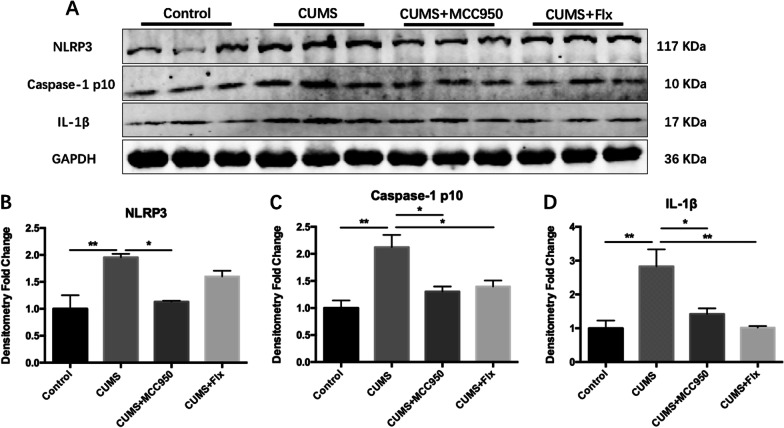
MCC950 inhibited CUMS-induced activation of NLRP3 inflammasome. **A** The expression of target protein was detected by Western Blotting. **B** MCC950 inhibited the up-regulation of NLRP3 expression induced by CUMS. **C** Both MCC950 and fluoxetine could significantly inhibit CUMS-induced activation of Caspase-1 p10. **D** CUMS induced up-regulation of IL-1β expression, and both MCC950 and fluoxetine could inhibit. The data were shown by mean and standard error (Mean ± SEM). (*n* = 3 for each group, *P < 0.05, **P < 0.01)

Compared with Control group, the expression of NLRP3 in hippocampus of CUMS group was remarkably increased in Fig. 5 (Fig. 5B; F_(3, 8)_ = 9.718, *P* = 0.0048; Tukey’s test: Control vs. CUMS, *P* = 0.0059), and the effector protein Caspase-1 p10 also increased significantly (Fig. 5C; F_(3, 8)_ = 9.979, *P* = 0.0044; Tukey’s test: Control vs. CUMS, *P* = 0.0033), followed by the increased expression of downstream product IL-1β (Fig. 5D; F _(3,8)_ = 8.829, *P* = 0.0064; Tukey’s test: Control vs. CUMS, *P* = 0.0092), suggesting that CUMS activated the expression of NLRP3 inflammasome. MCC950 obviously reduced the expression of the above indexes and inhibited the activation of inflammasome (Fig. 5B, Tukey’s test: CUMS vs. CUMS + MCC 950, *P* = 0.0140; Fig. 5C, CUMS vs. CUMS + MCC950, *P* = 0.0204; Fig. 5D, CUMS vs. CUMS + MCC950, *P* = 0.0368). Fluoxetine could not inhibit the expression of NLRP3, but could down-regulate the expression of Caspase-1 p10 and IL-1β (Fig. 5C, Tukey’s test: CUMS vs. CUMS + Flx, *P* = 0.0371; Fig. 5D, CUMS vs. CUMS + Flx, *P* = 0.0097).

### MCC950 inhibited the changes of Aβ metabolic pathway induced by CUMS

In order to further investigate the effect of CUMS on Aβ metabolic pathway, we took the protein from hippocampus of mice and made immunohistochemical staining to clarify the changes of Aβ metabolic pathway.

**Fig. 6:**
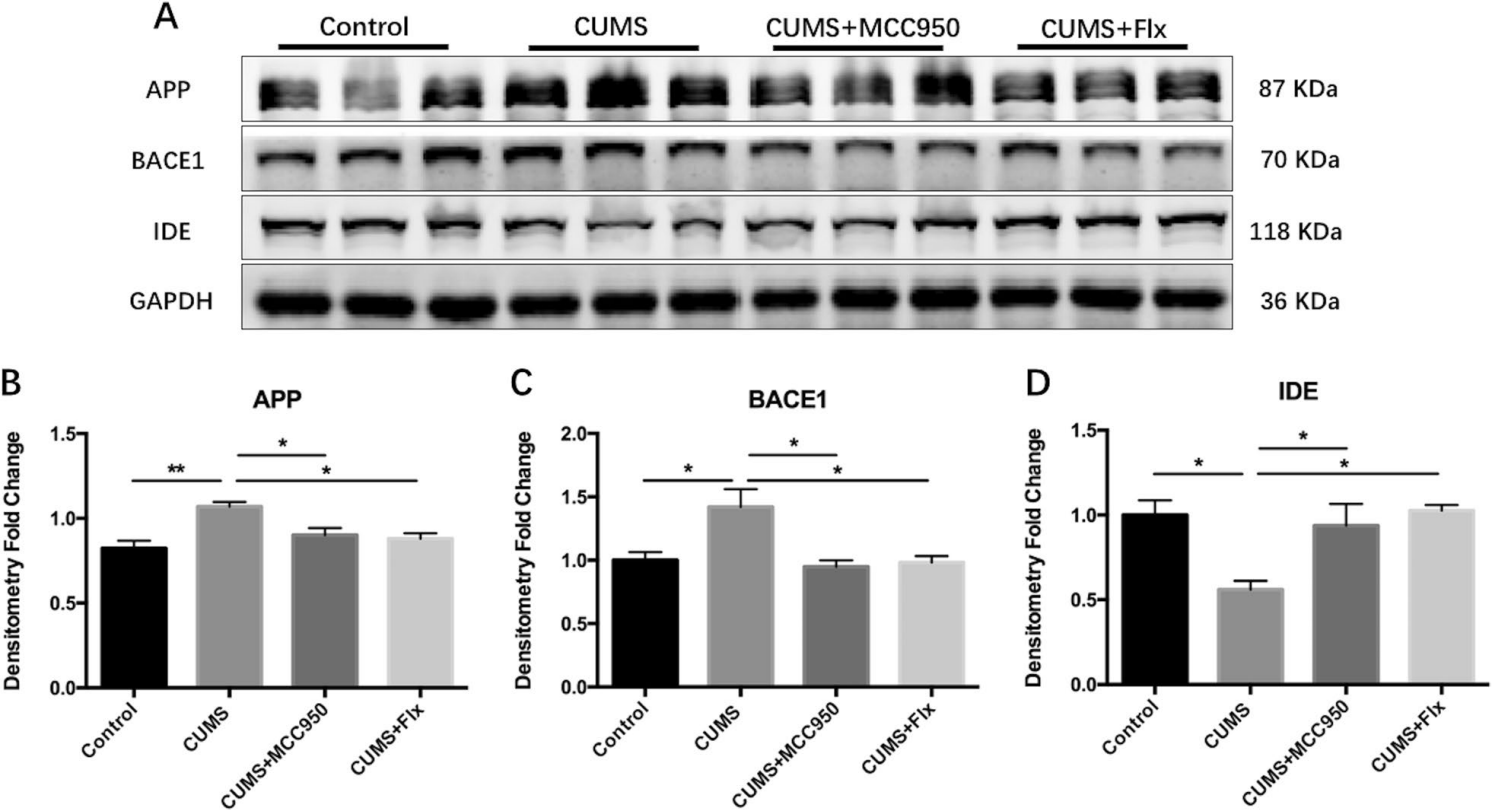
MCC950 restored CUMS-induced activation of Aβ metabolic pathway. **A** The expression of target protein was detected by Western Blotting. **B** Both MCC950 and fluoxetine could inhibit CUMS induced up-regulation of APP. **C** Both MCC950 and fluoxetine could relieve the increased expression of BACE1 induced by CUMS. **D** CUMS induced down-regulation of IDE, which could be improved by MCC950 and fluoxetine intervention. The data were shown by mean and standard error (Mean ± SEM). (*n* = 3 for each group, *P < 0.05, **P < 0.01)

As shown in Fig. 6, CUMS significantly upregulated the expression of β-amyloid precursor protein (APP), and both MCC950 and fluoxetine inhibited the upregulation of APP (Fig. 6B; F_(3, 8)_ = 8.222, *P* = 0.0079; Tukey’s test: Control vs. CUMS, *P* = 0.0066; CUMS vs. CUMS + MCC950, *P* = 0.0495; CUMS vs. CUMS + Flx, *P* = 0.0278). Compared with the control group, the expression of β-site amyloid precursor protein cleavage enzyme 1 (BACE1) in CUMS group increased significantly, while that of mice treated with MCC950 or fluoxetine decreased significantly compared with CUMS group (Fig. 6C; F_(3, 8)_ = 6.909, *P* = 0.0130; Tukey’s test: Control vs. CUMS, *P* = 0.0326; CUMS vs. CUMS + MCC950, *P* = 0.0182; CUMS vs. CUMS + Flx, *P* = 0.0265). The expression of insulin degrading enzyme (IDE) in CUMS group was obviously lower than that in control group, and MCC950 and fluoxetine could restrain the decrease of IDE expression induced by CUMS (Fig. 6D; F_(3, 8)_ = 6.894, *P* = 0.0131; Tukey’s test: Control vs. CUMS, *P* = 0.0231; CUMS vs. CUMS + MCC950, *P* = 0.0481; CUMS vs. CUMS + Flx, *P* = 0.0169).

Then, we carried out immunohistochemical staining to determine the deposition of amyloid β-protein in mice of each group. We selected the same sections of hippocampus in each group for comparison to reduce the error. As is indicated in Additional file 2: Fig. S1, in the hippocampus of mice in all groups, only a few mice showed a small amount of amyloid β-protein deposition. 5-week CUMS did not significantly increase the probability of β-amyloid protein deposition in mice.

### MCC950 inhibited the increase of tau protein phosphorylation induced by CUMS

Neurofibrillary tangles are another characteristic pathological manifestation of AD. Phosphorylated tau protein is the main component of neurofibrillary tangles. The main phosphorylation sites include Ser396, Ser404, Ser199, Ser202, and Thr231 [29]. In this study, Ser396 and Ser202 sites were applied to explore the effect of CUMS on tau protein phosphorylation.

**Fig. 7:**
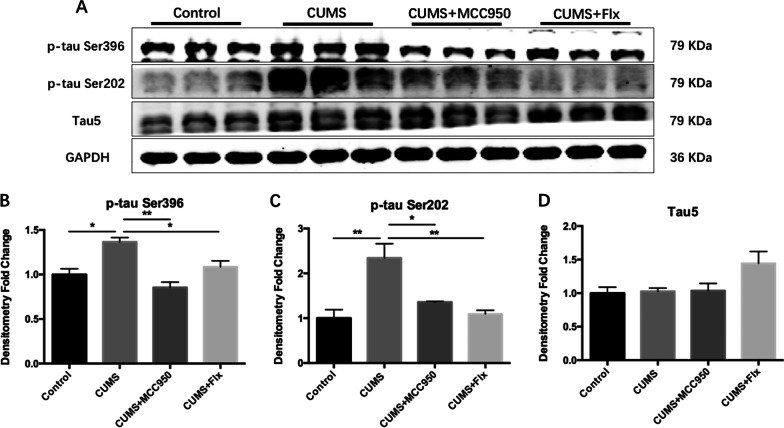
MCC950 inhibited the increase of tau protein phosphorylation induced by CUMS. **A** The expression of target protein was detected by Western Blotting. **B** Both MCC950 and fluoxetine could alleviate CUMS induced the phosphorylation of tau protein at Ser396 site. **C** Both MCC950 and fluoxetine could alleviate CUMS induced the phosphorylation of tau protein at Ser202 site. **D** There was no significant difference in Tau5 protein among the groups. The data were shown by mean and standard error (Mean ± SEM). (*n* = 3 for each group, *P < 0.05, **P < 0.01)

Compared with the control group, the phosphorylation level of tau protein in hippocampus of CUMS group mice was significantly higher at Ser396 and Ser202 sites (Fig. 7B; F_(3, 8)_ = 12.88, *P* = 0.0020; Tukey’s test: Control vs. CUMS, *P* = 0.0109; Fig. 7C; F_(3, 8)_ = 10.72, *P* = 0.0036; Tukey’s test: Control vs. CUMS, *P* = 0.0043). Compared with CUMS group, both MCC950 and fluoxetine could reduce the phosphorylation level of Ser396 and Ser202 sites of tau protein (Fig. 7B; Tukey’s test: CUMS vs. CUMS + MCC950, *P* = 0.0014; CUMS vs. CUMS + Flx, *P* = 0.0438; Fig. 7C; Tukey’s test: CUMS vs. CUMS + MCC950, *P* = 0.0249; CUMS vs. CUMS + Flx, *P* = 0.0068). There was no significant difference in the expression of Tau5 among the groups (Fig. 7D), indicating that there was no statistical difference in the level of total tau protein.

**Fig. 8:**
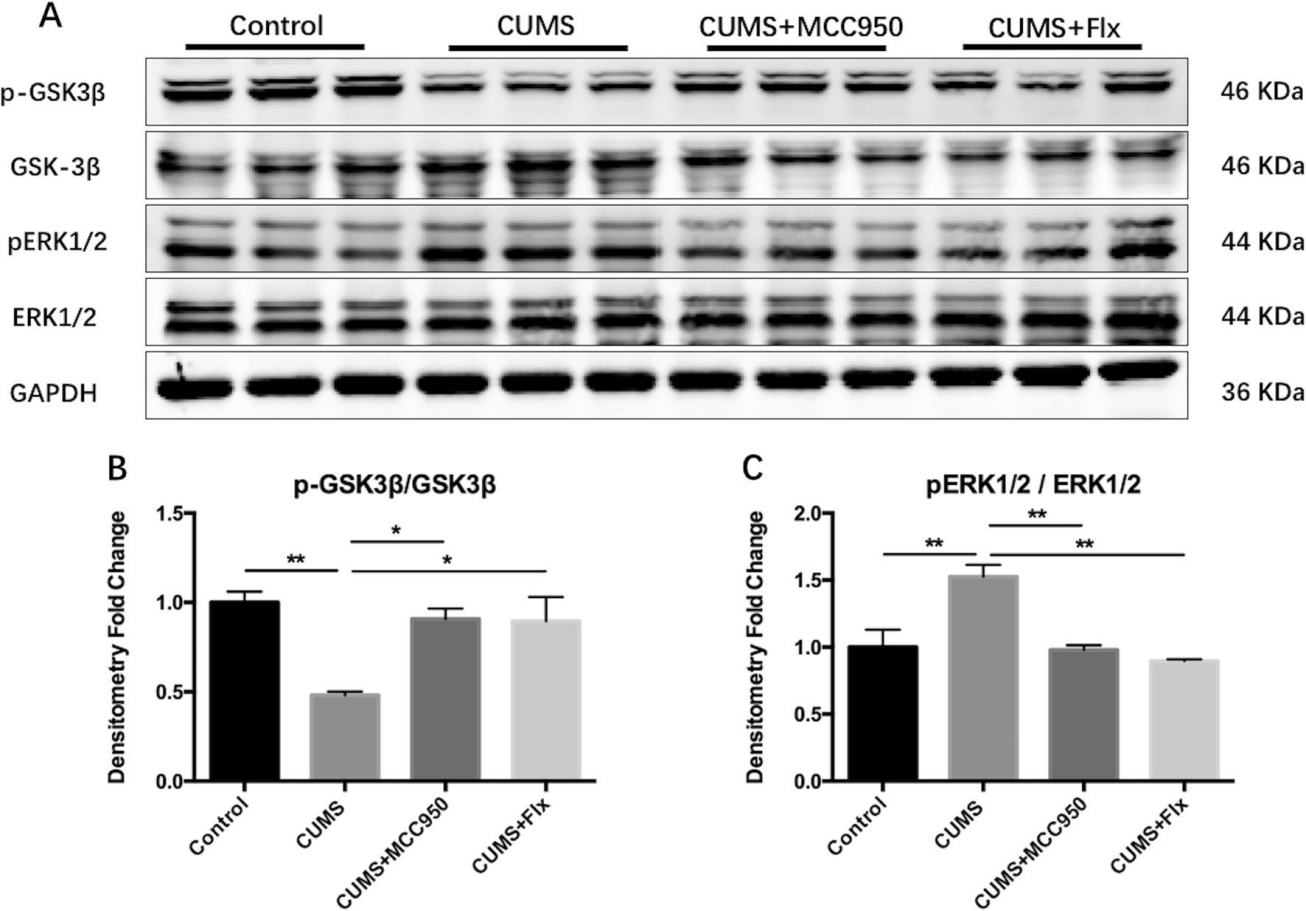
MCC950 reduced CUMS-induced activation of tau protein phosphorylation-related protein kinase. **A** The expression of target protein was detected by Western Blotting. **B** MCC950 and fluoxetine could improve CUMS induced the phosphorylation level of GSK-3β at Ser9 site. **C** CUMS significantly increased the phosphorylation level of ERK1/2 at Thr202/Tyr204 site, and both MCC950 and fluoxetine could decrease. The data were shown by mean and standard error (Mean ± SEM). (*n* = 3 for each group, *P < 0.05, **P < 0.01)

The phosphorylation level of tau protein is highly correlated with the activation of related protein kinases. As shown in Fig. 8, in comparison with the control group, the phosphorylation level of Glycogen synthase kinase-3β (GSK-3β) in hippocampus of CUMS group decreased obviously (Fig. 8B; F_(3, 8)_ = 8.430, *P* = 0.0074; Tukey’s test: Control vs. CUMS, *P* = 0.0076), while the phosphorylation level of Extracellular signal-regulated kinase 1/2 (ERK1/2) was significantly higher (Fig. 8C; F_(3, 8)_ = 12.46, *P* = 0.0022; Tukey’s test: Control vs. CUMS, *P* = 0.0079). As for MCC950 and fluoxetine groups, the phosphorylation level of GSK-3β increased (Fig. 8B; Tukey’s test: CUMS vs. CUMS + MCC950, *P* = 0.0221; CUMS vs. CUMS + Flx, *P* = 0.0258), while the phosphorylation level of ERK1/2 decreased comparing with control group (Fig. 8C; Tukey’s test: CUMS vs. CUMS + MCC950, *P* = 0.0063; CUMS vs. CUMS + Flx, *P* = 0.0026). In this study, the phosphorylation site of GSK-3β was Ser9, and the phosphorylation site of ERK1/2 was Thr202/Tyr204.

### LPS activated the expression of NLRP3 inflammasome in BV2 microglia in vitro

**Fig. 9:**
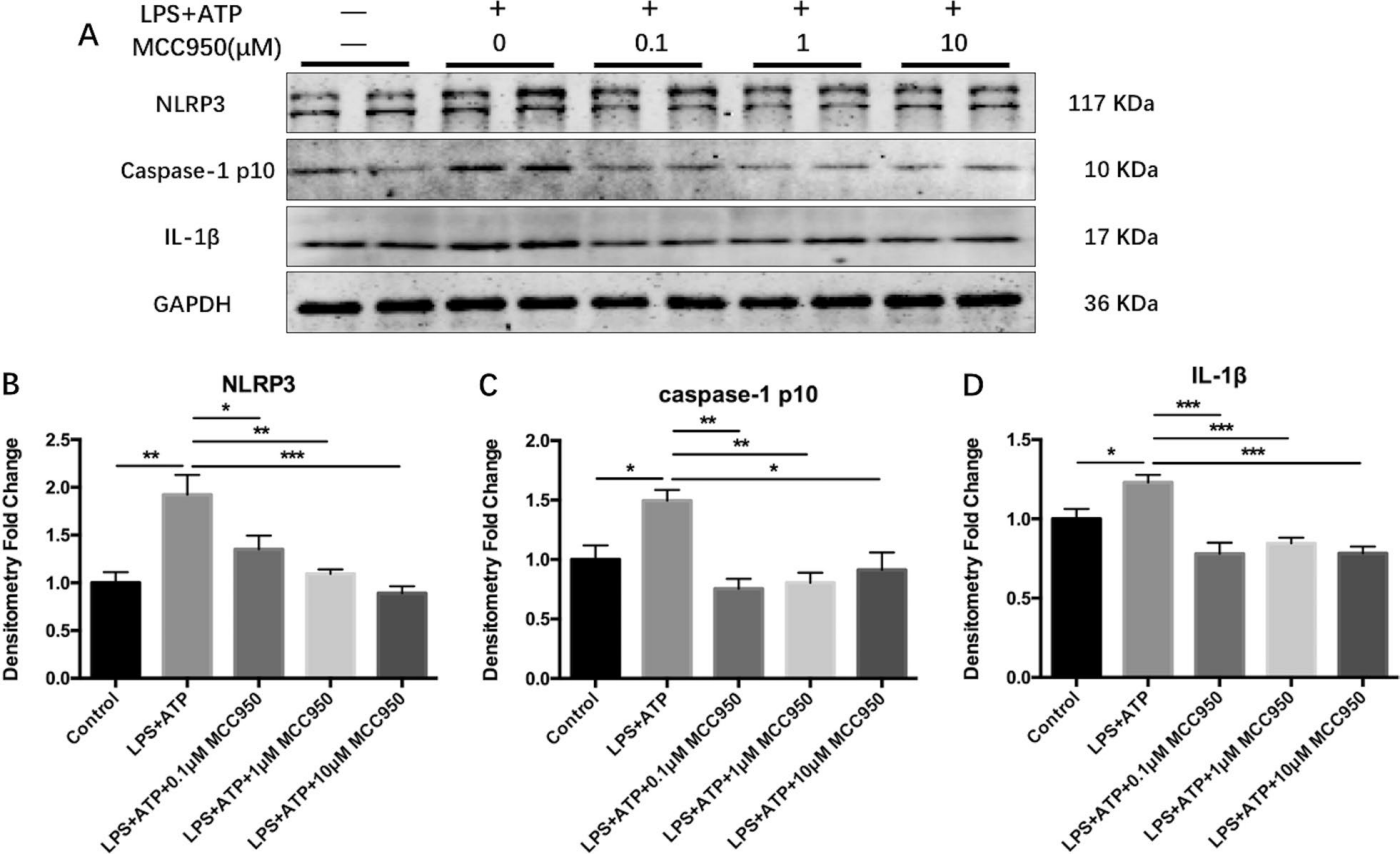
MCC950 ameliorated the activation of NLRP3 inflammasome in BV2 cells. **A** The expression of target protein was detected by Western Blotting. **B** MCC950 inhibited the up-regulation of NLRP3 induced by LPS + ATP. **C** MCC950 inhibited the activation of Caspase-1 p10 induced by LPS + ATP. **D** MCC950 alleviated the up-regulation of IL-1β induced by LPS + ATP. The data were shown by mean and standard error (Mean ± SEM). (*n* = 4 for each group, *P < 0.05, **P < 0.01, ***P < 0.001)

As exhibited in Fig. 9, LPS (1 μg/mL) + ATP (1 mM) stimulation up-regulated the level of NLRP3 (Fig. 9B; F_(4, 15)_ = 10.29, *P* = 0.0003; Tukey’s test: Control vs. LPS + ATP, *P* = 0.0011) and the expression of Caspase-1 p10 (Fig. 9C; F_(4, 15)_ = 7.566, *P* = 0.0015; Tukey’s test: Control vs. LPS + ATP, *P* = 0.0367) in BV2 cells. While 0.1 μM MCC950, 1 μM MCC950, and 10 μM MCC950 could inhibit the expression of NLRP3 (Fig. 9B; Tukey’s test: LPS + ATP vs. LPS + ATP + 0.1 μM MCC950, *P* = 0.0445; LPS + ATP vs. LPS + ATP + 1 μM MCC950, *P* = 0.0030; LPS + ATP vs. LPS + ATP + 10 μM MCC950, *P* = 0.0004) and Caspase-1 p10 (Fig. 9C; Tukey’s test: LPS + ATP vs. LPS + ATP + 0.1 μM MCC950, *P* = 0.0016; LPS + ATP vs. LPS + ATP + 1 μM MCC950, *P* = 0.0032; LPS + ATP vs. LPS + ATP + 10 μM MCC950, *P* = 0.0121). Correspondingly, LPS + ATP could also up-regulate the expression of IL-1β, the downstream product of NLRP3 inflammasome (Fig. 9D; F_(4, 15)_ = 13.08, *P* < 0.0001; Tukey’s test: Control vs. LPS + ATP, *P* = 0.0497). Three different concentrations of MCC950 could inhibit the up-regulation of IL-1β (Fig. 9D; Tukey’s test: LPS + ATP vs. LPS + ATP + 0.1 μM MCC950, *P* = 0.0002; LPS + ATP vs. LPS + ATP + 1 μM MCC950, *P* = 0.0010; LPS + ATP vs. LPS + ATP + 10 μM MCC950, *P* = 0.0002).

### LPS activated the expression of Aβ metabolism-related proteins in BV2 microglia

**Fig. 10:**
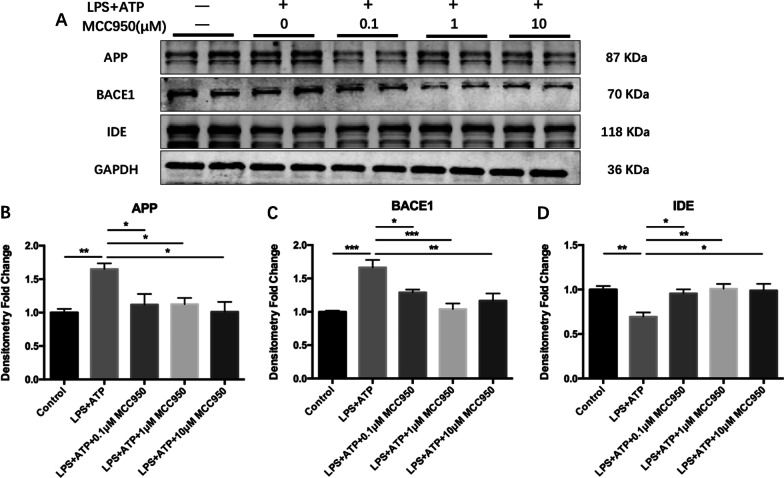
MCC950 alleviated the activation of Aβ metabolic pathway in BV2 cells. **A** The expression of target protein was detected by Western Blotting. **B** MCC950 inhibited the up-regulation of APP in BV2 cells induced by LPS + ATP. **C** MCC950 ameliorated the up-regulation of BACE1 induced by LPS + ATP. **D** MCC950 improved the down-regulation of IDE induced by LPS + ATP. The data were shown by mean and standard error (Mean ± SEM). (*n* = 4 for each group, *P < 0.05, **P < 0.01, ***P < 0.001)

As demonstrated in Fig. 10, LPS + ATP induced the increase of β-amyloid precursor protein (APP) in microglia (Fig. 10B; F_(4, 15)_ = 5.410, *P* = 0.0067; Tukey’s test: Control vs. LPS + ATP, *P* = 0.0090), 0.1 μM MCC950, 1 μM MCC950 and 10 μM MCC950 could all reduce the expression of APP (Fig. 10B; Tukey’s test: LPS + ATP vs. LPS + ATP + 0.1 μM MCC950, *P* = 0.0370; LPS + ATP vs. LPS + ATP + 1 μM MCC950, *P* = 0.0395; LPS + ATP vs. LPS + ATP + 10 μM MCC950, *P* = 0.0103). Furthermore, LPS + ATP significantly up-regulated the expression of BACE1 (Fig. 10C; F_(4, 15)_ = 10.63, *P* = 0.0003; Tukey's test: Control vs. LPS + ATP, *P* = 0.0003), and decreased the expression of IDE (Fig. 10D; F_(4, 15)_ = 6.067, *P* = 0.0041; Tukey’s test: Control vs. LPS + ATP, *P* = 0.0087). The intervention of three different concentrations of MCC950 could inhibit the expression of BACE1 (Fig. 10C; Tukey’s test: LPS + ATP vs. LPS + ATP + 0.1 μM MCC950, *P* = 0.0374; LPS + ATP vs. LPS + ATP + 1 μM MCC950, *P* = 0.0006; LPS + ATP vs. LPS + ATP + 10 μM MCC950, *P* = 0.0049) and improve the level of IDE (Fig. 10D; Tukey’s test: LPS + ATP vs. LPS + ATP + 0.1 μM MCC950, *P* = 0.0279; LPS + ATP vs. LPS + ATP + 1 μM MCC950, *P* = 0.0068; LPS + ATP vs. LPS + ATP + 10 μM MCC950, *P* = 0.0115).

### Activated BV2 microglia promoted tau protein phosphorylation in primary neurons

We extracted primary mouse neurons to observe the changes of tau protein after activation of NLRP3 inflammasome. Additional file 3: Fig. S2 showed that the purity of mouse primary neurons was over 95% as verified by neuron specific marker NeuN.

**Fig. 11:**
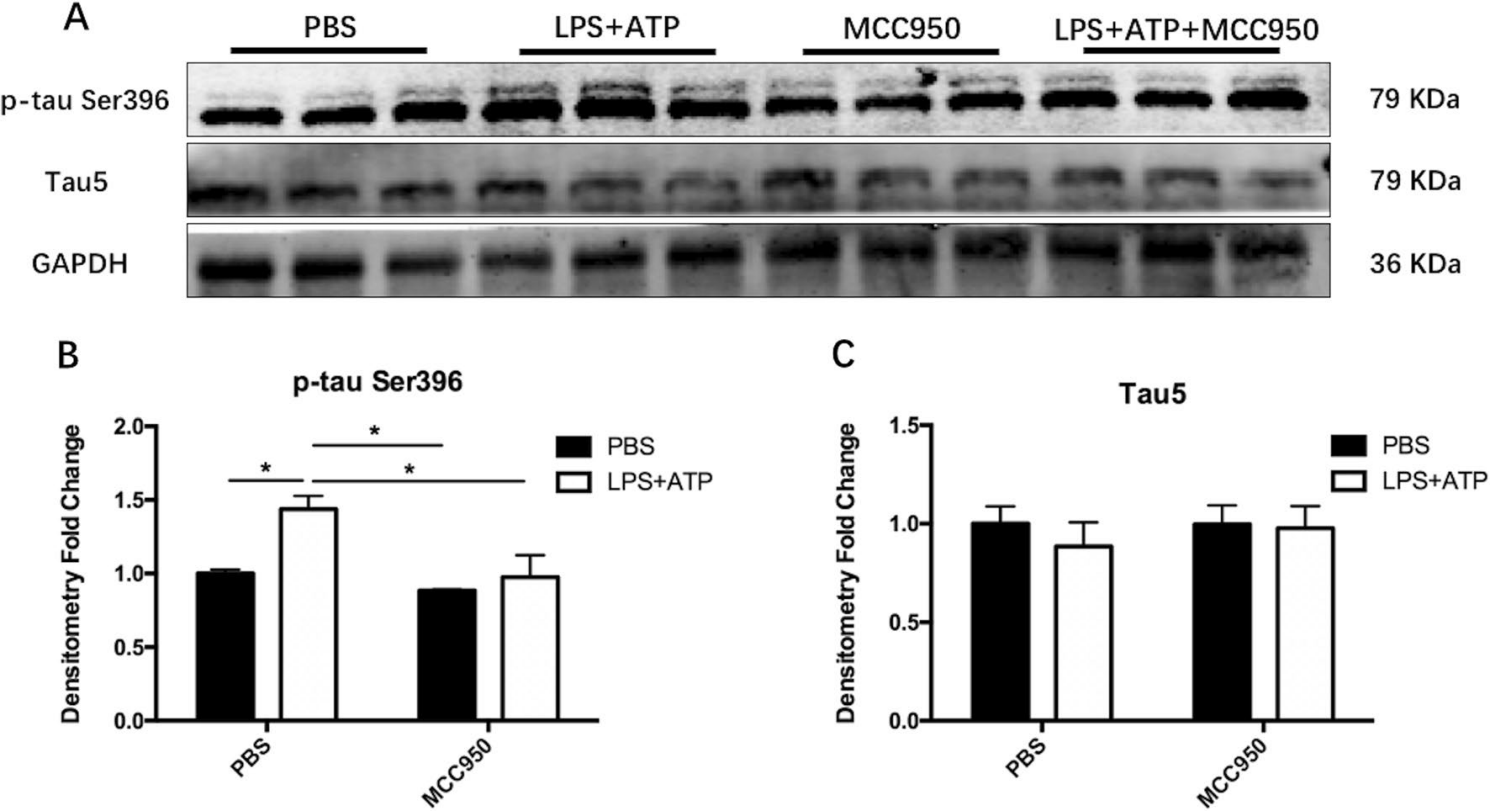
MCC950 reduced the phosphorylation level of tau protein. **A** The expression of target protein was detected by Western Blotting. **B** The secretion of BV2 cells activated by LPS + ATP induced the up-regulation of the phosphorylation level of tau protein in Ser396 site, and MCC950 could inhibit it. **C** The secretion of BV2 cells activated by LPS + ATP had no influence on the expression of total tau protein. The data were shown by mean and standard error (Mean ± SEM). (*n* = 3 for each group, *P < 0.05)

As shown in Fig. 11, secretions produced by LPS + ATP activating BV2 cells could up-regulate the phosphorylation level of primary neuron tau protein at Ser396 site (Fig. 11B; LPS + ATP: F_(1, 8)_ = 10.95, *P* = 0.0107; MCC950: F_(1, 8)_ = 9.165, *P* = 0.0164; Bonferroni’s test: Control vs. LPS + ATP, *P* = 0.0456). When 1 μM MCC950 was administrated to BV2 cells before LPS + ATP stimulation, the cell secretion could significantly inhibit the activation of neuron tau protein at Ser396 site (Fig. 11B; Bonferroni’s test: LPS + ATP vs. LPS + ATP + MCC950, *P* = 0.0342). There is no significant difference in the expression level of total tau protein Tau5 among groups (Fig. 11C).

**Fig. 12:**
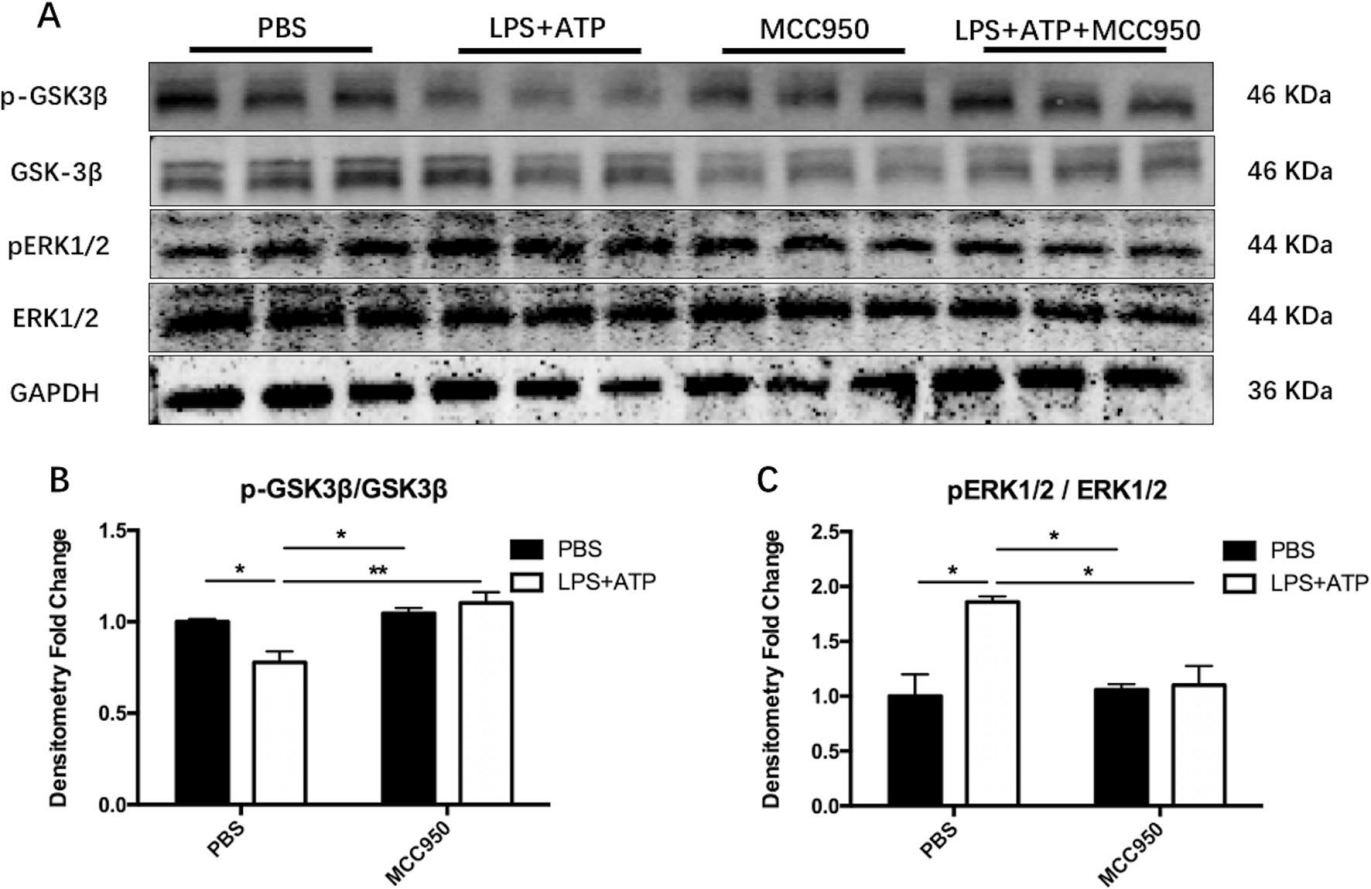
MCC950 reduced the activation of protein kinase related to tau protein phosphorylation. **A** The expression of target protein was detected by Western Blotting. **B** MCC950 inhibited the down-regulation of GSK-3β phosphorylation in primary neurons induced by the secretion of BV2 cells activated by LPS + ATP. **C** The secretion of BV2 cells activated by LPS + ATP induced the up-regulation of ERK1/2 phosphorylation in primary neurons, and the intervention of MCC950 could alleviate. The data were shown by mean and standard error (Mean ± SEM). (*n* = 3 for each group, *P < 0.05, **P < 0.01)

Then, we detected the activity of protein kinase. The secretion of BV2 cells stimulated by LPS + ATP could down-regulate the phosphorylation level of GSK-3β at Ser9 site of neurons (Fig. 12B; LPS + ATP: F_(1, 8)_ = 16.87, *P* = 0.0034; MCC950: F_(1, 8)_ = 3.49, *P* = 0.0987; Bonferroni’s test: Control vs. LPS + ATP, *P* = 0.0494), and up-regulate the phosphorylation level of neuron ERK1/2 at Thr202/Tyr204 (Fig. 12C; LPS + ATP: F_(1, 8)_ = 6.504, *P* = 0.0342; MCC950: F_(1, 8)_ = 10.87, *P* = 0.0109; Bonferroni’s test: Control vs. LPS + ATP, *P* = 0.0129). Intervention with 1 μM MCC950 before LPS + ATP stimulation could significantly increase the phosphorylation level of GSK-3β in neurons (Fig. 12B; Bonferroni’s test: LPS + ATP vs. LPS + ATP + MCC 950, *P* = 0.0058), and decrease ERK1/2 phosphorylation level (Fig. 12C; Bonferroni’s test: LPS + ATP vs. LPS + ATP + MCC950, *P* = 0.0265).

## Discussion

Although a large number of epidemiological studies have shown that stress is an important risk factor for cognitive decline and event AD, the underlying mechanism remains unclear [30]. Previous studies have shown that chronic stress can induce inflammatory reaction by activating NLRP3 inflammasome, thus contribute to depressive-like behaviors in mice [19]. Neuro-inflammation is a common pathophysiological feature of depression and AD. Depression is accompanied by chronic inflammatory changes and promotes the development of AD [31]. Therefore, the activation of NLRP3 induced by chronic stress may be a potential mechanism of cognitive decline in depression.

Consistent with previous studies, CUMS induced depressive-like behaviors and cognitive decline in mice [32, 33]. Several studies have proved that inhibiting the activity of NLRP3 inflammasome can alleviate the depressive-like behaviors of mice. Present findings further confirmed that the cognitive function of CUMS mice can be improved by inhibiting NLRP3 inflammasome. Chronic stress promoted the activation of NLRP3 inflammasome and microglia, which was the major source of CNS inflammatory cytokines [34]. MCC950 could effectively inhibit not only the expression of NLRP3 inflammasome, but also the activation of microglia.

At the same time, we observed the changes of neurons in hippocampus of mice. DCX is generally considered as a marker of immature neurons [35]. After 5 weeks of CUMS, the number of DCX positive cells in hippocampus of mice in stress group decreased significantly, indicating that chronic stress can damage immature neurons in hippocampus. MCC950 intervention could protect immature neurons, but fluoxetine had no obvious effect. In addition, as previously reported, the number of Nissl staining positive cells in CA1 and CA3 areas of hippocampus of CUMS group mice decreased significantly, indicating that slow stress caused damage to the structure and function of neurons, and even decreased the number of neurons. However, the intervention of MCC950 and fluoxetine could play a good therapeutic effect, and significantly increased the number of Nissl positive fine cells in CA1 and CA3 regions.

Another interesting aspect of this study was the APP misprocessing and tau phosphorylation of mice induced by chronic stress. The imbalance of Aβ homeostasis led to the accumulation and aggregation of Aβ outside the cell [36]. Our results showed that CUMS significantly increased the expression of β-amyloid precursor protein (APP) and β-secretase BACE1, and down-regulated the expression of Aβ-degrading enzyme IDE, thus affecting the normal metabolism of Aβ pathway. However, no obvious plaque deposition was found in the hippocampus of mice in each group. A previous study had some similar results. The study has shown that CUMS disturbs the process of APP and up-regulates the accumulation of Aβ, but has no effect on plaque deposition [37]. This meant that the cognitive decline caused by CUMS may be a physiological change in the early stage of AD, and increasing the duration or intensity of stress may increase the probability of plaque deposition induced by chronic stress. MCC950 could improve Aβ metabolic pathway disorder induced by CUMS and reduce cognitive dysfunction caused by Aβ deposition.

Tau protein is a microtubule binding protein. Hyper-phosphorylated tau protein would separate from microtubules, and gather in the form of neurofibrillary tangles in neuronal cell bodies and neurites [38]. Our study showed that CUMS up-regulated the phosphorylation level of tau protein at Ser396 and Ser202 sites, but the total tau protein level did not change. It has been reported that CUMS can induce the phosphorylation level of tau protein in hippocampus of rodents to increase at Ser396, Ser404, Ser199, Ser202, and Thr231 and other sites [29], which may be an important mechanism of depression as a risk factor for AD. The high phosphorylation level of tau protein required the regulation of GSK-3β, ERK1/2 and other protein kinases. MCC950 could reduce the phosphorylation level of tau protein and promote the recovery of cognitive function by regulating the activation of protein kinases, such as GSK-3β and ERK1/2.

The results of in vitro were consistent with in vivo experiments, which further confirmed the role of NLRP3 inflammasome in cognitive decline in depression. Evidence showed that numerous microglia gather near the Aβ plaque of AD patients. Microglia played a dual role in the pathogenesis of Aβ [39]. On the one hand, microglia helped to eliminate Aβ aggregation through phagocytosis. On the other hand, microglia could promote the accumulation of Aβ by releasing neurotoxic proteases and inflammatory factors [40]. The activation of NLRP3 inflammasome reduced the phagocytosis of Aβ by microglia, thus increasing the deposition of Aβ and promoting the development of AD [41]. Furthermore, microglia expressed a large number of APP isomers. The activation of microglia could increase the expression of APP [42]. Microglial activation would also increase the phosphorylation level of tau protein [43]. Under the influence of activated microglia, many signal molecules, such as phosphatase, GSK-3, ERK1/2, etc., were involved in the pathological changes of tau protein [44]. Therefore, Carroll et al. proposed that microglia activation is an important reason for the pathological changes of tau protein [43]. This study demonstrated that pretreatment of microglia with NLRP3 inflammasome inhibitor MCC950 could restore Aβ metabolic pathway to normal and decrease tau protein phosphorylation level. NLRP3 inflammasome may affect the cognitive function of depression patients through these mechanisms.

Some limitations remained in the present study. First, after 5 weeks of CUMS, no plaque deposition was found in hippocampus of stressed mice. This was probably because the stress time is not long enough and the stress intensity is relatively small. In future experiments, we can raise mice for a little longer and observe if they are more likely to suffer from AD when they are old. Second, in this study, only a few phosphorylation sites of tau protein in mice were detected. We are supposed to focus on the sites of hyper-phosphorylated tau protein in cerebrospinal fluid of patients with depression, and compare the sites in patients with different degrees of depression in future. Third, the in vivo concentration of MCC950 we chose may not be optimum. According to the previous studies, the effective in vivo dose of MCC950 ranged from 1 mg/kg to 20 mg/kg [45, 46]. We chose the dose of 10 mg/kg, which was similar to the study of Liu et al. [47]. We hope the following studies can clarify the optimal concentration of MCC950.

In summary, as far as we know, our research has identified for the first time the key role of NLRP3 inflammasome in the adverse effects of stress on cognitive function. At the same time, the activation of NLRP3 inflammasome promoted the disorder of Aβ metabolism and the high phosphorylation of tau protein, which might also be an important reason for depression as a risk factor for AD.

## Conclusion

Collectively, the present study highlights new findings on the important role of NLRP3 inflammasome in the development of cognitive decline in depression. Inhibition of NLRP3 inflammasome could alleviate CUMS-induced depressive-like behaviors and cognitive decline in mice, and inhibit the activation of AD physiological indicators. This study deepened the understanding of the relationship between cognitive decline in depression and AD and provided a new target for the treatment of cognitive decline in depression.

## Supplementary Information

**Additional file 1: Table S1.** Primary antibodies used in Western Blotting.

**Additional file 2: Figure S1.** Aβ deposition in hippocampus of mice.

**Additional file 3: Figure S2.** The purity of primary mouse neurons.

## Data Availability

All data generated in this work are included in this manuscript.

## Abbreviations

AD: Alzheimer’s disease
CUMS: Chronic unpredictable mild stress
IL-1β: Interleukin-1β
SPT: Sucrose preference test
TST: Tail suspension test
MWM: Morris water maze
ATCC: American Type Culture Collection
LPS: Lipopolysaccharides
ATP: Adenosine triphosphate
DCX: Doublecortin
APP: β-Amyloid precursor protein
BACE1: β-Site amyloid precursor protein cleavage enzyme 1
IDE: Insulin-degrading enzyme
GSK-3β: Glycogen synthase kinase-3β
ERK1/2: Extracellular signal-regulated kinase 1/2

## References

[1] Yang L, Zhao Y, Wang Y, Liu L, Zhang X, Li B, Cui R (2015). The effects of psychological stress on depression. Curr Neuropharmacol.

[2] Ray A, Gulati K, Rai N (2017). Stress, anxiety, and immunomodulation: a pharmacological analysis. Vitam Horm.

[3] Malhi GS, Mann JJ (2018). Depression. Lancet.

[4] Ahern E, Semkovska M (2017). Cognitive functioning in the first-episode of major depressive disorder: a systematic review and meta-analysis. Neuropsychology.

[5] Chakrabarty T, Hadjipavlou G, Lam RW (2016). Cognitive dysfunction in major depressive disorder: assessment, impact, and management. Focus (Am Psychiatr Publ).

[6] Stotland NL (2012). Recovery from depression. Psychiatr Clin North Am.

[7] Sáiz-Vázquez O, Gracia-García P, Ubillos-Landa S, Puente-Martínez A, Casado-Yusta S, Olaya B, Santabárbara J (2021). Depression as a risk factor for Alzheimer’s disease: a systematic review of longitudinal meta-analyses. J Clin Med.

[8] Newcombe EA, Camats-Perna J, Silva ML, Valmas N, Huat TJ, Medeiros R (2018). Inflammation: the link between comorbidities, genetics, and Alzheimer’s disease. J Neuroinflammation.

[9] DiSabato DJ, Sheridan JF (2021). Stress, inflammation and depression: a new possible molecular pathway. Brain Behav Immun.

[10] Jin K, Lu J, Yu Z, Shen Z, Li H, Mou T, Xu Y, Huang M (2020). Linking peripheral IL-6, IL-1β and hypocretin-1 with cognitive impairment from major depression. J Affect Disord.

[11] Shen XN, Niu LD, Wang YJ, Cao XP, Liu Q, Tan L, Zhang C, Yu JT (2019). Inflammatory markers in Alzheimer’s disease and mild cognitive impairment: a meta-analysis and systematic review of 170 studies. J Neurol Neurosurg Psychiatry.

[12] Lynch MA (2015). Neuroinflammatory changes negatively impact on LTP: a focus on IL-1β. Brain Res.

[13] Hueston CM, Cryan JF, Nolan YM (2017). Stress and adolescent hippocampal neurogenesis: diet and exercise as cognitive modulators. Transl Psychiatry.

[14] Schroder K, Tschopp J (2010). The inflammasomes. Cell.

[15] Ward R, Li W, Abdul Y, Jackson L, Dong G, Jamil S, Filosa J, Fagan SC, Ergul A (2019). NLRP3 inflammasome inhibition with MCC950 improves diabetes-mediated cognitive impairment and vasoneuronal remodeling after ischemia. Pharmacol Res.

[16] Fu Q, Wu J, Zhou XY, Ji MH, Mao QH, Li Q, Zong MM, Zhou ZQ, Yang JJ (2019). NLRP3/Caspase-1 pathway-induced pyroptosis mediated cognitive deficits in a mouse model of sepsis-associated encephalopathy. Inflammation.

[17] Liu R, Tang W, Wang W, Xu F, Fan W, Zhang Y, Zhang C (2021). NLRP3 influences cognitive function in schizophrenia in Han Chinese. Front Genet.

[18] Zhang Y, Liu L, Peng YL, Liu YZ, Wu TY, Shen XL, Zhou JR, Sun DY, Huang AJ, Wang X (2014). Involvement of inflammasome activation in lipopolysaccharide-induced mice depressive-like behaviors. CNS Neurosci Ther.

[19] Zhang Y, Liu L, Liu YZ, Shen XL, Wu TY, Zhang T, Wang W, Wang YX, Jiang CL (2015). NLRP3 inflammasome mediates chronic mild stress-induced depression in mice via neuroinflammation. Int J Neuropsychopharmacol.

[20] Liu LL, Li JM, Su WJ, Wang B, Jiang CL (2019). Sex differences in depressive-like behaviour may relate to imbalance of microglia activation in the hippocampus. Brain Behav Immun.

[21] Li JM, Liu LL, Su WJ, Wang B, Zhang T, Zhang Y, Jiang CL (2019). Ketamine may exert antidepressant effects via suppressing NLRP3 inflammasome to upregulate AMPA receptors. Neuropharmacology.

[22] Wang B, Huang X, Pan X, Zhang T, Hou C, Su WJ, Liu LL, Li JM, Wang YX (2020). Minocycline prevents the depressive-like behavior through inhibiting the release of HMGB1 from microglia and neurons. Brain Behav Immun.

[23] Wu D, Chen Y, Sun Y, Gao Q, Li H, Yang Z, Wang Y, Jiang X, Yu B (2020). Target of MCC950 in inhibition of NLRP3 inflammasome activation: a literature review. Inflammation.

[24] Li P, Li L, Yu B, Wang X, Wang Q, Lin J, Zheng Y, Zhu J, He M, Xia Z (2021). Doublecortin facilitates the elongation of the somatic Golgi apparatus into proximal dendrites. Mol Biol Cell.

[25] Shan X, Chen J, Dai S, Wang J, Huang Z, Lv Z, Wang Q, Wu Q (2020). Cyanidin-related antidepressant-like efficacy requires PI3K/AKT/FoxG1/FGF-2 pathway modulated enhancement of neuronal differentiation and dendritic maturation. Phytomedicine.

[26] Kádár A, Wittmann G, Liposits Z, Fekete C (2009). Improved method for combination of immunocytochemistry and Nissl staining. J Neurosci Methods.

[27] Wasilewska B, Najdzion J, Szteyn S (2002). The neuronal structure of the globus pallidus in the rabbit–Nissl and Golgi studies. Folia Morphol (Warsz).

[28] Mandwie M, Piper JA, Gorrie CA, Keay KA, Musumeci G, Al-Badri G, Castorina A (2022). Rapid GFAP and Iba1 expression changes in the female rat brain following spinal cord injury. Neural Regen Res.

[29] Reimer L, Betzer C, Kofoed RH, Volbracht C, Fog K, Kurhade C, Nilsson E, Överby AK, Jensen PH (2021). PKR kinase directly regulates tau expression and Alzheimer’s disease-related tau phosphorylation. Brain Pathol.

[30] Machado A, Herrera AJ, de Pablos RM, Espinosa-Oliva AM, Sarmiento M, Ayala A, Venero JL, Santiago M, Villarán RF, Delgado-Cortés MJ (2014). Chronic stress as a risk factor for Alzheimer’s disease. Rev Neurosci.

[31] Caraci F, Copani A, Nicoletti F, Drago F (2010). Depression and Alzheimer’s disease: neurobiological links and common pharmacological targets. Eur J Pharmacol.

[32] Jia Z, Yang J, Cao Z, Zhao J, Zhang J, Lu Y, Chu L, Zhang S, Chen Y, Pei L (2021). Baicalin ameliorates chronic unpredictable mild stress-induced depression through the BDNF/ERK/CREB signaling pathway. Behav Brain Res.

[33] Han B, Yu L, Geng Y, Shen L, Wang H, Wang Y, Wang J, Wang M (2016). Chronic stress aggravates cognitive impairment and suppresses insulin associated signaling pathway in APP/PS1 mice. J Alzheimers Dis.

[34] Ransohoff RM, Brown MA (2012). Innate immunity in the central nervous system. J Clin Invest.

[35] Hanson ND, Owens MJ, Nemeroff CB (2011). Depression, antidepressants, and neurogenesis: a critical reappraisal. Neuropsychopharmacology.

[36] Sadigh-Eteghad S, Sabermarouf B, Majdi A, Talebi M, Farhoudi M, Mahmoudi J (2015). Amyloid-beta: a crucial factor in Alzheimer’s disease. Med Princ Pract.

[37] Xie F, Zhao Y, Ma J, Gong JB, Wang SD, Zhang L, Gao XJ, Qian LJ (2016). The involvement of homocysteine in stress-induced Aβ precursor protein misprocessing and related cognitive decline in rats. Cell Stress Chaperones.

[38] Kadavath H, Hofele RV, Biernat J, Kumar S, Tepper K, Urlaub H, Mandelkow E, Zweckstetter M (2015). Tau stabilizes microtubules by binding at the interface between tubulin heterodimers. Proc Natl Acad Sci U S A.

[39] Grathwohl SA, Kälin RE, Bolmont T, Prokop S, Winkelmann G, Kaeser SA, Odenthal J, Radde R, Eldh T, Gandy S (2009). Formation and maintenance of Alzheimer’s disease beta-amyloid plaques in the absence of microglia. Nat Neurosci.

[40] Cai Z, Hussain MD, Yan LJ (2014). Microglia, neuroinflammation, and beta-amyloid protein in Alzheimer’s disease. Int J Neurosci.

[41] Bai H, Zhang Q (2021). Activation of NLRP3 inflammasome and onset of Alzheimer’s disease. Front Immunol.

[42] Gu SM, Park MH, Hwang CJ, Song HS, Lee US, Han SB, Oh KW, Ham YW, Song MJ, Son DJ, Hong JT (2015). Bee venom ameliorates lipopolysaccharide-induced memory loss by preventing NF-kappaB pathway. J Neuroinflammation.

[43] Carroll JC, Iba M, Bangasser DA, Valentino RJ, James MJ, Brunden KR, Lee VM, Trojanowski JQ (2011). Chronic stress exacerbates tau pathology, neurodegeneration, and cognitive performance through a corticotropin-releasing factor receptor-dependent mechanism in a transgenic mouse model of tauopathy. J Neurosci.

[44] Pooler AM, Polydoro M, Wegmann S, Nicholls SB, Spires-Jones TL, Hyman BT (2013). Propagation of tau pathology in Alzheimer’s disease: identification of novel therapeutic targets. Alzheimers Res Ther.

[45] Dong Y, Li S, Lu Y, Li X, Liao Y, Peng Z, Li Y, Hou L, Yuan Z, Cheng J (2020). Stress-induced NLRP3 inflammasome activation negatively regulates fear memory in mice. J Neuroinflammation.

[46] Xu X, Huang X, Zhang L, Huang X, Qin Z, Hua F (2021). Adiponectin protects obesity-related glomerulopathy by inhibiting ROS/NF-κB/NLRP3 inflammation pathway. BMC Nephrol.

[47] Liu Q, Zhang MM, Guo MX, Zhang QP, Li NZ, Cheng J, Wang SL, Xu GH, Li CF, Zhu JX, Yi LT (2022). Inhibition of microglial NLRP3 with MCC950 attenuates microglial morphology and NLRP3/Caspase-1/IL-1β signaling in stress-induced mice. J Neuroimmune Pharmacol.

