# Supplementary material for: The involvement of NLRP3 inflammasome in CUMS-induced AD-like pathological changes and related cognitive decline in mice
Source: J Neuroinflammation. 2023 May 10;20:112. doi: 10.1186/s12974-023-02791-0 (PMC10173607; doi:10.1186/s12974-023-02791-0)
Supplement: Supplementary file 1 — Additional file 1: Table S1. Primary antibodies used in Western Blotting. [file 12974_2023_2791_MOESM1_ESM.docx]

**Table S1** Primary antibodies used in Western Blotting.

| **Primary antibody** | **Dilution** | **Host species** | **Cat#** | **Supplier** |
| --- | --- | --- | --- | --- |
| GFAP | 1:1000 | Mouse | 3670S | Cell Signaling |
| Iba1 | 1:1000 | Rabbit | ab178846 | Abcam |
| NLRP3 | 1:1000 | Rabbit | ab214185 | Abcam |
| Caspase-1 p10 | 1:1000 | Rabbit | sc-514 | Santa Cruz |
| IL-1β | 1:800 | Rabbit | ab9722 | Abcam |
| APP | 1:1000 | Rabbit | ab32136 | Abcam |
| BACE1 | 1:1000 | Rabbit | 5606S | Cell Signaling |
| IDE | 1:1000 | Rabbit | ab133561 | Abcam |
| Tau5 | 1:1000 | Mouse | ab80579 | Abcam |
| p-tau Ser396 | 1:1000 | Rabbit | ab109390 | Abcam |
| p-tau Ser202 | 1:1000 | Rabbit | 39357S | Cell Signaling |
| GSK-3β | 1:1000 | Rabbit | 22104-1-AP | Proteintech |
| pGSK-3β | 1:1000 | Rabbit | 9323S | Cell Signaling |
| ERK1/2 | 1:1000 | Rabbit | 4695S | Cell Signaling |
| pERK1/2 | 1:1000 | Rabbit | 8544S | Cell Signaling |
| GAPDH | 1:2000 | Rabbit | 10494-1-AP | Proteintech |
