# Supplementary figures and images for: The involvement of NLRP3 inflammasome in CUMS-induced AD-like pathological changes and related cognitive decline in mice
Source: J Neuroinflammation. 2023 May 10;20:112. doi: 10.1186/s12974-023-02791-0 (PMC10173607; doi:10.1186/s12974-023-02791-0)

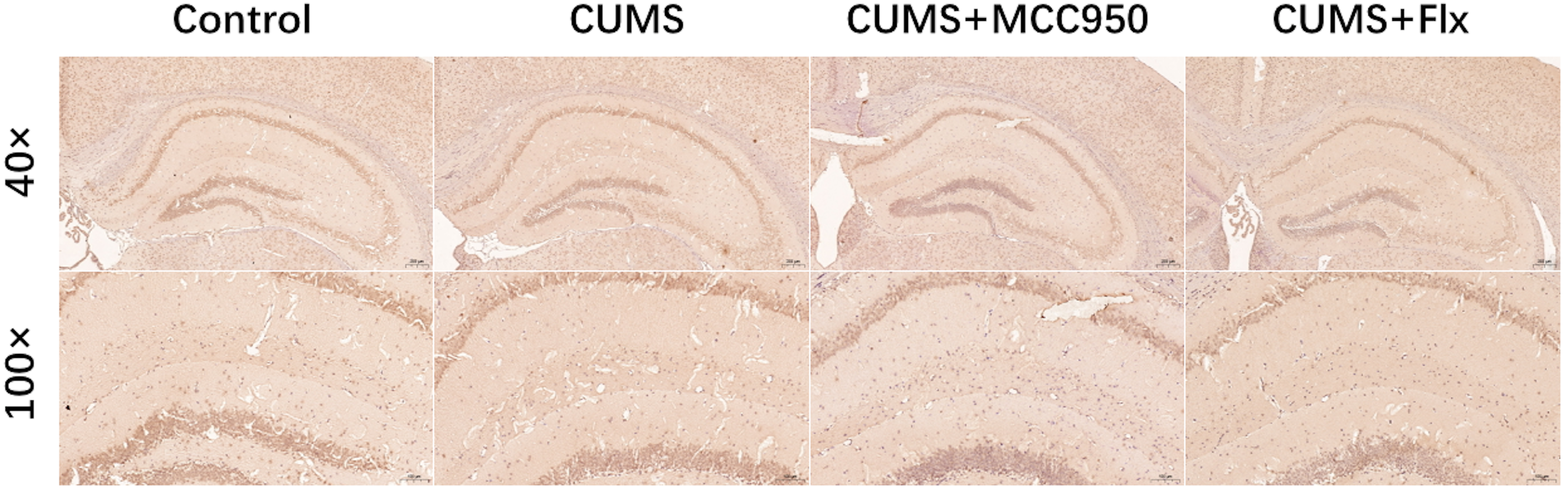

Supplement: Supplementary file 2 — Additional file 2: Figure S1. Aβ deposition in hippocampus of mice. [file 12974_2023_2791_MOESM2_ESM.jpg]

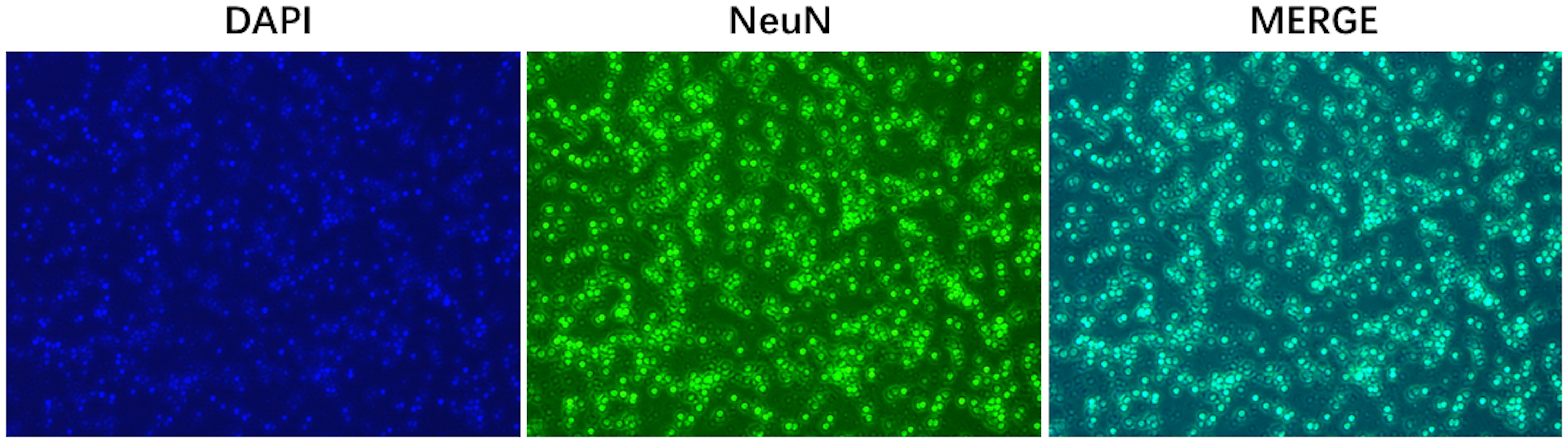

Supplement: Supplementary file 3 — Additional file 3: Figure S2. The purity of primary mouse neurons. [file 12974_2023_2791_MOESM3_ESM.jpg]
